# The Potential Role of Cytokines in Diabetic Intervertebral Disc Degeneration

**DOI:** 10.14336/AD.2022.0129

**Published:** 2022-10-01

**Authors:** Sunlong Li, Chongan Huang, Jian Xiao, Yuhao Wu, Zengjie Zhang, Yifei Zhou, Naifeng Tian, Yaosen Wu, Xiangyang Wang, Xiaolei Zhang

**Affiliations:** ^1^Department of Orthopaedics, The Second Affiliated Hospital and Yuying Children’s Hospital of Wenzhou Medical University, Wenzhou, Zhejiang, China.; ^2^Key Laboratory of Orthopaedics of Zhejiang Province, Wenzhou, Zhejiang, China.; ^3^The Second School of Medicine, Wenzhou Medical University, Wenzhou, Zhejiang, China.; ^4^Chinese Orthopaedic Regenerative Medicine Society, Hangzhou, Zhejiang, China.; ^5^Department of Orthopedic Surgery, The Second Affiliated Hospital, Zhejiang University School of Medicine, Hangzhou, Zhejiang, China.; ^6^Orthopedics Research Institute of Zhejiang University, Hangzhou, Zhejiang, China.; ^7^Key Laboratory of Motor System Disease Research and Precision Therapy of Zhejiang Province, Hangzhou, Zhejiang, China

**Keywords:** intervertebral disc degeneration (IVDD), Diabetes, cytokines

## Abstract

Intervertebral disc degeneration (IVDD) is a major cause of low back pain. Diabetes mellitus is a chronic inflammatory disease that may cause or aggravate IVDD; however, the mechanism by which diabetes induce IVDD is currently unclear. Compared to non-diabetic individuals, diabetic patients have higher levels of plasma cytokines, especially TNF-α, IL-1β, IL-5, IL-6, IL-7, IL-10, and IL-18. Due to the crucial role of cytokines in the process of intervertebral disc degeneration, we hypothesized that elevation of these cytokines in plasma of diabetic patients may be involved in the process of diabetes-induced IVDD. In this review, changes in plasma cytokine levels in diabetic patients were summarized and the potential role of elevated cytokines in diabetes-induced IVDD was discussed. Results showed that some cytokines such as TNF-α and IL-1β may accelerate the development of IVDD, while others such as IL-10 is supposed to prevent its development. Apoptosis, senescence, and extracellular matrix metabolism were found to be regulated by these cytokines in IVDD. Further studies are required to validate the cytokines targeted strategy for diabetic IVDD therapy.

Globally, low back pain (LBP) is a common chronic disease that is associated with a significant financial burden [1]. Approximately 40% of the global population suffers from LBP during their lifetime [2]. In the US, it has been reported that from 1996 to 2013, low back and neck pain-associated treatment costs increased by an estimated $87.6 billion. Among the Chinese people, LBP is the leading cause of disability [3, 4]. The potential causes of LBP involve the pathological changes of the spinal column, including intervertebral disc and facet joints. Intervertebral disc degeneration (IVDD), which has a high global incidence, is a major risk factor for low back pain [5]. Clinically, IVDD is a complicated disease that is caused by multiple factors, including age, genetic and environmental factors. It is characterized by biochemical and cellular changes in the disc tissue [6-9]. Occupational habits such as heavy lifting and lifestyle habits like lack of exercises and driving cars for extended periods of time contribute to IVDD development [10]. Moreover, smoking and trauma are associated with the pathogenesis of IVDD [8-11]. However, the mechanisms involved in IVDD have not been clearly established.

## The pathogenesis of intervertebral disc degeneration

The intervertebral disc (IVD), an elegant structure, is composed of nucleus pulposus (NP), annulus fibrosus (AF) and cartilage endplate (CEP). The gelatinous nucleus pulposus, which is predominantly composed of Type II collagen (Col II) and proteoglycans with a high-water content, is the major component of IVD. It is important in counteracting physiological stress due to human activities [12]. In addition, nucleus pulposus is important in stabilization and biomechanical maintenance of the disc. Nucleus pulposus cell necrosis and apoptosis is highly associated with degeneration of intervertebral discs [13]. The loss of nucleus pulpous impairs the balance between extracellular matrix (ECM) synthesis and degradation, leading to IVDD development. Clinically, IVDD is a degenerative disease that is mainly common among the elderly. Thus, studies have investigated the relationship between senescence and IVDD [14, 15]. Consistent with these studies, we found that nucleus pulposus cell senescence accelerated IVDD progression, which was ameliorated when NP cell senescence was inhibited [16, 17]. Therefore, NP cell apoptosis and senescence is involved in IVDD. Annulus fibrosus, a component of the intervertebral disc tissue, is a thick and dense structure, including the inner and outer annulus. It protects the nucleus pulposus by alleviating nucleus pulposus and vertebral body stress. During IVDD, collagen II levels gradually decrease, leading to annular disruption and IVDD progression [18]. The nucleus pulposus and annulus fibrosus play a significant role in IVDD progression. Moreover, the cartilage endplate is involved in IVDD progression. Due to its restrictive blood supply effects, the endplate is crucial in disc degeneration [19, 20]. Cartilage endplate apoptosis and senescence blocks blood nutrition supply and initiates IVDD [20]. IVDD is a complicated pathological process that involves various factors, such as nutrition, cell senescence, apoptosis, inflammation, cytokines and extracellular matrix degradation of the disc tissue. The definite etiology and pathophysiology of IVDD should be further investigated.

## Association between diabetes mellitus and intervertebral disc degeneration

Diabetes mellitus, which is associated with elevated blood glucose levels, is a chronic disease. It results from insulin deficiency and insulin resistance and is classified as type 1 diabetes (T1D) or type 2 diabetes (T2D). In 2013, the estimated overall prevalence of diabetes was 10.9% while that of prediabetes was 35.7% among adults in China [21]. Diabetes mellitus-associated complications, including neuropathy, nephropathy and cardiovascular diseases, significantly decrease the quality of life and increase mortality rates [22]. There is an association between diabetes mellitus and IVDD. First, Sakellaridis reported a significant increase in lumber disc surgery incidences among diabetes mellitus patients [23]. In Finland, a study involving 638 diabetic and 32510 non-diabetic individuals revealed that herniated disc incidences in diabetic patients were significantly higher than in non-diabetic patients [24]. In Asia, a 4-year case follow-up study in Japan showed that diabetes was closely associated with upper lumbar disc degeneration (OR=6.83; 95% CI, 1.07 -- 133.7) [25]. A retrospective study reported that being an immune disease with an early onset time and difficult glucose control, T1D results in early IVDD [26]. Don-Kyu Kim et al. [27] documented that T2D is significantly associated with degenerative lumbar spine disorders, therefore, they postulated that diabetes is a predisposing factor for lumbar spine disorders. In our previous study, to exclude the effects of other interfering factors, we used animal models to evaluate the effects of diabetes on IVDD alone. We established that nucleus pulposus cell senescence and apoptosis in STZ-induced diabetic rats were markedly increased while the extracellular matrix was degraded, leading to IVDD [28]. It has also been suggested that T1D contributes to IVDD by promoting aggrecan degradation and apoptosis [29, 30]. In a previous study, T2D induced by leptin receptor-deficient knockout (db/db) led to IVDD by elevating MMP3 levels and promoting cell apoptosis [31]. *In vivo* and *in vitro* studies have supported the hypothesis that diabetes is a major risk factor for IVDD. Therefore, we defined diabetes-induced IVDD as Diabetic Intervertebral Disc Degeneration (DB-IVDD). Although clinical and animal studies have confirmed that diabetes can cause or worsen IVDD, the exact pathomechanisms have not been conclusively determined. Given the increasing diabetes incidences, it is important to investigate the potential pathomechanisms of diabetes-induced IVDD.

## Mechanisms of diabetes-induced intervertebral disc degeneration

Apoptosis, senescence, advanced glycation end products (AGEs) accumulation, microvascular damage, changes in the extracellular matrix (ECM) and direct impairment by hyperglycemia have been associated with diabetes-induced IVDD. Cell death and ECM degradation are major mechanisms of IVDD, including diabetes-induced IVDD. Cell death, a functional biological process required for cellular development, is classified as apoptosis, necrosis or autophagy [32]. Cell death modulation is associated with various diseases and highly contributes to IVDD [33]. The main intervertebral disc components are collagen II and aggrecans. Due to intrinsic regulation by growth and catabolic factors, anabolism and catabolism of ECM are in equilibrium [18]. When the balance is broken, IVDD development is initiated. Studies have principally focused on regulation of apoptosis, the autophagic pathway and ECM degradation in IVDD, including in diabetes-induced IVDD.

High glucose, reactive oxygen species (ROS), accumulation of AGEs, inflammation and obesity are major factors that contribute to diabetes-induced IVDD [34, 35]. Diabetes-induced degenerative changes have been associated with decreased endplate porosity, increased thickness, and accumulation of advanced glycation end products (AGEs) [36]. In STZ-induced diabetic rats, AGEs accumulation in the nucleus pulposus accelerated disc degeneration by upregulating the levels of matrix degrading enzymes (MMP-2) [35]. In addition, AGEs accumulation in NP may promote disc degeneration-associated inflammation by disturbing the extracellular matrix via NLRP3 inflammasome activation [37]. An experimental study also suggested that AGEs induce AF cells apoptosis, which may provide a theoretical basis for diabetic IVD degeneration [38]. Chronic ingestion of AGEs has a significant effect of IVDD [39]. Mechanistically, AGEs may also enhance endochondral ossification in intervertebral discs, thereby aggravating IVDD. Endplate cartilage calcification plays a significant role in accelerating disc degeneration by blocking nutritional supply [40].

Elevated blood glucose levels, a major characteristic of diabetes mellitus-related disease, have a direct or indirect influence on disc degeneration. High glucose upregulates ROS levels, which promotes the apoptosis of NP as well as CEP cells and enhances the catabolic activities of the ECM, aggravating IVDD [41, 42]. The pathogenesis of IVDD is tightly associated with ROS [43]. ROS modulates homeostasis through various signaling pathways, including the nuclear factor-κB (NF-κB) pathway, the mitogen-activated protein kinases (MAPKS) pathway, and the PI3K/AKT pathway [44]. Hyperglycemia-induced ROS promotes NP cell apoptosis through the mitochondrial apoptosis pathway [45]. The death of notochordal cells through the mitochondrial apoptosis and death receptor pathways marks the induction of IVDD [46]. Senescence, a cellular state that is characterized by cell cycle arrest, can be accelerated by oxygen free radicals’ accumulation. The p53/p21 and p16/pRB pathways are the two major pathways that modulate the senescent state. These two pathways are modulated by various factors, including oxidative stress and inflammation [47]. ROS-induced mitochondrial dysfunction and oxidative stress contributes to disc cell senescence, thereby promoting IVDD. High glucose affects the viability of nucleus pulposus cells and matrix degrading enzymes [48]. Moreover, high glucose significantly modulates the expressions of ECM-related proteins, including TIMPs downregulation and MMPs overexpression (1, 3, 13), resulting in rapid IVDD and fibrosis. Collagen II and proteoglycans were found to be suppressed in high glucose treated NP cells [36, 41]. Since the endplate cartilage is the main route for nutrition supply, cartilage endplate degeneration can block nutrition supply [49]. Nutritional deprivation leads to cell death and ECM degradation, which triggers IVDD [49]. Excess apoptosis and calcification of cartilaginous endplate cells accelerates cartilage endplate degeneration [20]. During IVDD, ROS is the main stimuli for cartilage endplate apoptosis and calcification [20]. Autophagy is a complicated process whose main function is to degrade damaged organelles and useless proteins [50]. Animal model studies reported that autophagy was enhanced by high blood glucose, which can be seen as a protective measure against apoptosis and senescence. Metformin protects nucleus pulposus cells from apoptosis and senescence by stimulating autophagy [16, 51]. The silent mating type information regulator 2 homolog1 (sirt1), an NAD+ dependent histone deacetylase is associated with various aging-related diseases and plays an important role in cellular senescence as well as disc cell apoptosis. Non-restriction of calories in the nucleus pulposus due to high glucose degrades sirt1 activities, which enhances disc cell apoptosis [52].

Diabetes mellitus is a chronic inflammatory disease. Inflammation plays an important role in IVDD pathogenesis [53]. In addition, circulating levels of acute-phase proteins as well as some cytokines have been shown to be elevated in diabetes patients [54, 55]. Hyperglycemia impairs β cell functions and directly or indirectly activates immune responses, thereby inducing the changes in levels of circulating cytokines and other proteins [54]. Vascular ingrowth in the nucleus pulposus is a vital pathological phenomenon of IVDD [56, 57]. LA Binch et al. [58] stated that cytokine secretion, particularly IL-1β, during IVDD progression facilitates vascular ingrowth, via which cytokines can exert their effectiveness, implying that vascular ingrowth suppression may be a potential therapeutic strategy [59]. However, the effects of dysregulated cytokine levels in DB-IVDD have not been conclusively determined.

## Potential roles of elevated cytokines in DB-IVDD

In this section, we elucidate on some cytokines that are differentially expressed between diabetic and healthy individuals (Table 1). Studies have reported that IL-6 is significantly elevated in both T1D and T2D (Table 1) and is a proven risk factor and an independent predictor for T2D [60-62]. Due to IL-6-induced HS-CRP production, plasma IL-1β levels have been shown to be elevated in T1D and T2D [55, 63]. However, recent studies have reported that differences in IL-1β levels between T2D and healthy people are not significant (p>0.05; Table 1) [60, 61]. Compared to healthy people, IL-10, an anti-inflammatory factor released to ameliorate inflammation, is elevated in T1D and T2D patients (Table 1) [64, 65]. Levels of IL-18, a cytokine in the interleukin-1 family that is involved in the development and progression of diet-induced cardiac dysfunctions, are elevated in T2D patients (Table 1) [66]. In addition, compared to healthy individuals, plasma TNF-α levels are significantly elevated in both types of diabetes patients (Table 1). As a pro-inflammatory cytokine, TNF-α may have an important role in IVDD [60]. Moreover, TNF-α levels are also increased in diabetes (Table 1), where it may enhance the production of inflammatory factors by T cells. Compared to healthy individuals, hepatocyte growth factor (HGF) and vascular endothelial growth factor receptor (VEGFR-1/2) levels are higher in diabetic patients (Table 1) [67]. In pre-diabetics, IL-5 and IL-7 levels were found to be elevated, compared to controls (Table 1). Elevations of IL-7 in T1D is a risk factor for diabetic nephropathy [68], which has potential associations with IL-5 [69]. Various factors have been attributed to the development of diabetic nephropathy, which may share similar mechanisms with IVDD [70]. Therefore, IL-5 and IL-7 may have a potential impact on IVDD.

**Table 1:** Plasma concentrations of some cytokines in diabetic patients and healthy individuals.

	Control	T1D	T2D	Prediabetes	p	Reference
**IL-1β(pg/ml)**	3.2	6.4			0.0104	[55]
**IL-1β(pg/ml)**	0.47±0.79		0.57±0.93		0.1959	[60]
**IL-2(pg/ml)**	4.2	7.6			0.0087	[55]
**IL-2R (pg/ml)**	50.80±7.269		121.4±22.75		0.049	[67]
**IL-4(pg/ml)**	9.37±2.98			12.42±2.86	0.32	[68]
**IL-5(pg/ml)**	0.40±0.11			1.19± 0.26	0.01	[68]
**IL-6(pg/ml)**	1.9±0.6	5.0±1.3			<0.02	[62]
**IL-6 (pg/ml)**	1.67±1.59		2.45±1.80		<0.0001	[60]
**IL-7(pg/ml)**	2.8	7.1			0.0034	[55]
**IL-7 (pg/ml)**	1.43±0.38			2.85±0.53	0.01	[68]
**IL-10(pg/ml)**	7.6	33.4			<0.05	[65]
**IL-10 (pg/ml)**	1.76±0.94		3.02±2.27		0.0163	[64]
**IL-16 (pg/ml)**	105.2±20.43		112.2±21.62		0.08198	[67]
**IL-18 (pg/ml)**	34.75±4.82		88.47±12.13		0.0073	[67]
**IFN-α(pg/ml)**	4.233±0.489		4.845±4.845		0.72	[67]
**IFN-γ (pg/ml)**	2.24±0.45			3.57±0.59	0.21	[68]
**TNF-α(pg/ml)**	11.3	24.2			<0.05	[55]
**TNF-α (pg/ml)**	1.79±1.28		2.03±1.51		<0.0094	[60]
**TNF-αR2(pg/ml)**	2383		2646.5		<0.001	[134]
**HGF (pg/ml)**	589.1±47.02		863.1±126.9		0.045	[67]
**VEGFR-1(pg/ml)**	643.8		2044		0.0001	[67]
**VEGFR-2 (pg/ml)**	7103		19190		0.0005	[67]
**sIL-6R (pg/ml)**	35040	35370			0.13	[135]
**SIL-6Ra (pg/ml)**	45900		65900		0.032	[67]

Data are displayed as median (IQR) or mean ±SEM. T1D: type 1 diabetes mellitus. T2D: type 2 diabetes mellitus.

Cytokines are important regulators of diabetes mellitus and IVDD [71]. As degeneration proceeds, elevated inflammatory cytokine levels accelerate the process of IVDD by enhancing aggrecan as well as collagen degradation and promoting phenotypic changes of disc cells [5]. Moreover, inflammatory cytokines can induce the death of disc cells and ECM degradation, thereby contributing to IVDD [53]. Diabetes is a chronic inflammatory disease that is associated with alterations in various inflammatory factors [72]. Several cytokines are elevated in diabetes patients, where they accelerate IVDD. Therefore, we summarized some of the elevated cytokines in diabetes and briefly discussed the mechanisms through which these cytokines accelerate IVDD in diabetics.

## TNF-α in DB-IVDD

Tumor necrosis factor (TNF-α), an important member of the TNF superfamily of ligands, secreted by macrophagocytes, T cells and some non-immune cells, is a pro-inflammatory cytokine. As previously mentioned, TNF-α is elevated in type 1 and 2 diabetes patients. Bachmeier reported that degenerative and herniated human IVD tissues have higher TNF-α levels, compared to non-degenerative IVD tissues [73]. Through cell apoptosis, senescence, autophagy, ECM degradation and inflammation, elevated TNF-α plays a significant role in DB-IVDD progression [74]. Evidence supports the hypothesis that TNF-α is involved in IVD cell apoptosis. TNF-α elevates the apoptotic rate and up-regulates p53 as well as cleaved-caspase 3 levels in various cells. [75]. Cytochrome C, which is involved in apoptosis, is also associated with TNF-α-induced IVDD [76]. TNF-α enhances apoptosis in IVDD by activating JNK/ERK-MAPK and NF-κB signaling pathways [77]. In addition, autophagy is involved in TNF-α-induced IVDD, as a catabolic mechanism against cell stress. Annulus fibrosus (AF) plays an important role in IVDD. After treatment with TNF-α, autophagy-related proteins, such as autophagy modulator p62 and WIPI49, in AF cells were increased, suggesting that TNF-α activates autophagy but, at the same time, blocks the autophagy flux [78]. Using mice models, Risbud found that systemic TNF-α elevation was not enough to promote complete disc degeneration, however, it caused spontaneous disc herniation [79]. Cheng Wang showed that TNF-α stimulated NP and AF cells to synthesize many pro-inflammatory cytokines, such as IL-6, IL-8, IL-1β, IL-8 and IL-17, thereby amplifying inflammatory responses in inflammation-induced IVDD [74]. Given that ECM degradation is tightly associated with DB-IVDD progression, TNF-α also promotes ECM degradation by inducing the production of various enzymes such as MMPs and ADAMTSs, which are responsible for ECM degradation,[74, 80, 81] we believe that TNF-a plays a facilitating role in DB-IVDD.

## IL-1β in DB-IVDD

IL-1β is a pro-inflammatory cytokine whose over-production exerts deleterious effects on peripheral insulin signaling and β-cell functions [82]. IL-1β is a critical effector molecule in non-obese diabetic (NOD) mice models of T1D, and it’s also an important inflammatory mediator of type II diabetes [83, 84]. Inflammatory responses are induced by overexpression of inflammatory cytokines, mainly IL-1β, and are highly involved in IVDD progression [85]. IL-1 stimulates the production of several metalloproteinases, leading to connective tissue breakdown and suppression of proteoglycan as well as type II collagen levels, thereby exerting a global negative effect on articular cartilage. In addition, IL-1 exerts direct and indirect stimulatory effects on osteoclast maturation, and therefore, participates in the development of bony erosions in arthritis [86]. Qiu-Hui Pan demonstrated that disc cells pre-treated with IL-1β increased their apoptotic rates in response to FasL *in vitro* [87]. Risbud MV et al. [88] concluded that IL-1β regulates SDC4 expressions, which play a key role in the pathogenesis of degenerative disc diseases by promoting aggrecan degradation via ADAMTS-5 in the nucleus pulposus. In our previous study, we found that IL-1β induced the expressions of senescence-associated secreted phenotype (SASP) factors, which might influence the microenvironment in NP tissues and lead to local dysfunctions [89]. Cao Yang reported that reactive oxygen species (ROS) induced NF-κB pathway activation promotes NLRP3 inflammasome activation and IL-1β release, both of which enhances NP degeneration [90]. Therefore, specifically elevated IL-1β in diabetes may promote the occurrence of DB-IVDD.

## IL-5 in DB-IVDD

IL-5 was first discovered as a "T-cell replacing factor" that is secreted by T cells to stimulate antibody production by B cells [91]. The major biological function of IL-5 is to promote eosinophil activation, proliferation and migration. It exerts its effects on target cells via the IL-5 receptor (IL-5R), which is composed of a unique α chain (IL-5R to CD125) and the common cytokine β-chain, which is essential for IL-5 signal transduction [92]. Immune responses in NP tissues lead to chronic inflammation and consistent pain in patients. Plasma IL-5 levels have been shown to be elevated in prediabetes patients, compared to healthy volunteers. However, the significance of IL-5 in IVDD has not been conclusively determined. As previously reported, JAK2 and STATs are indispensable in IL-5 dependent signaling transduction in B cells, Th2 cells and eosinophils [93]. JAK2 and STATs signaling pathways are also involved in IVDD progression [94], suggesting that IL-5 participates in IVDD (including DB-IVDD) via the JAK2/STATs signaling pathway. Moreover, Ras GTPase extracellular signaling pathways are involved in IL-5 dependent cell death, proliferation and differentiation of eosinophils [93, 95]. Ras is also associated with extracellular matrix degradation in the chondrocytes, suggesting that IL-5 influences Collagen II and MMPs levels in intervertebral disc tissues in IVDD [96, 97]. Polarization of helper T lymphocytes (Th2) may be involved in IVDD via phenotypic shifts of macrophages [98], and IL-5-induced eosinophils can activate macrophages [99], suggesting that IL-5 influences IVDD progression through various immune responses in the disc tissue. Thus, the significance of IL-5 in DB-IVDD should be investigated.

## IL-6 in DB-IVDD

IL-6, a 25KDa protein, has a characteristic structure that is made up of four long α-helices arranged in an up-up-down-down topology. It is a cytokine that promotes B cell maturation into antibody producing cells [100]. IL-6 plays a great role in various physiological functions as well as in immune regulation [101]. Moreover, it stimulates osteoclast formation and promotes bone resorption [102]. Signal transduction is activated by IL-6 via IL-6R and sIL-6R, which contains the signal-transducing component (gp130). Binding of IL-6 to the IL-6R/gp130 complex primarily signals through JAK/STAT, Ras and PI3K pathways and its function varies from growth and differentiation of B- and T- cells to acute-phase protein induction [53]. Many stimuli that activate IL-6 are associated with oxidative stress and damage [103]. IL-6 levels in circulating blood were found to be elevated in acute hyperglycemia, high fat meals, physical activity and before/after surgery [104]. Plasma IL-6 levels were found to be elevated in type 1 and 2 diabetes and were associated with T2D development [60]. Moreover, IL-6 was highly expressed in degenerative discs, causing low back pain and presenting both pro-inflammatory and anti-inflammatory functions [105, 106]. It regulates inflammatory responses by downregulating the levels of pro-inflammatory cytokines and upregulating anti-inflammatory molecules, including IL-1 receptor antagonist protein, TNF-soluble receptor and extrahepatic protease inhibitors [107]. Inhibition of STAT alleviates the effects of IL-6 in the intervertebral disc, therefore, the IL-6/JAK/STAT3 pathway is a potential therapeutic target for IVDD [108]. IL-6 suppresses H_2_O_2_-induced cell death by elevating prohibiting levels, which is involved in cell apoptosis and senescence [109]. Moreover, IL-6 promotes the expressions of proteins involved in IVDD, including COX-2 and MMP13. Higher ratios of IL-6/IL-10 plasma levels augments the risk of causing symptomatic lumbar osteoarthritis and IVDD [105]. Therefore, IL-6 plays a complex role in DB-IVDD progression.

## IL-7 in DB-IVDD

IL-7 is a member of the common γ chain (γc-CD132) cytokine family, which includes IL-2, IL-4, IL-9, IL-15 and IL-21 [110]. In pre-diabetics and T1D, IL-7 levels were increased, compared to the control group. IL-7 stimulates Janus kinase (JAK) and STAT signaling pathways, which subsequently activate the PI3K/Akt pathway to facilitate target gene transcriptions [110]. Even though IL-7 is rarely reported in IVDD, IL-5, IL-6, IL-7, IL-8 and MCP-2 were established to be significantly elevated in injured-IVD, compared to non-injury-IVD [111]. Senescence contributes to the development of various degenerative diseases, including osteoarthritis and IVDD [112, 113]. In cord blood cells, IL-7 increased telomere length and hTERT gene expressions [114], suggesting that it may also protect against cellular senescence in other degenerative diseases. Elevated IL-7 enhances MMP-13 production in osteoarthritis patients, which has a significant effect in degenerative diseases [115]. It has been reported that IL-7 has comparable pathological characteristics in osteoarthritis and IVDD. However, the significance of IL-7 in IVDD is unknown. The JAK/STAT signaling pathway may be mechanistically involved in IL-7-related IVD degeneration. This is because, IL-7 stimulates the secretion of S100A4, which has been verified to be elevated in osteoarthritis and upregulates the expressions of MMP13 by activating the JAK/STAT pathway [116]. In addition, IL-7 can be stimulated by other cytokines, such as IL-1 and IL-6, and combine with other cytokines like TNF-α and IL-6 to exert its effects in IVDD [117]. However, the specific roles of IL-7 in DB-IVDD have not been conclusively determined.

## IL-10 in DB-IVDD

Interleukin-10, which is secreted by type 2 T-helper (Th2) cell clones, belongs to the IL-10 family of cytokines, including IL-19, IL-20, IL-22, IL-24, and IL-26 [118]. IL-10, an anti-inflammatory cytokine, is a protective factor in various tissues, including the articular cartilage and disc tissues. It inhibits innate and acquired immune responses by suppressing the activities of monocytes and the development of activated T-cells. Moreover, IL-10 modulates the functions and differentiation of various immune cells, such as B-cells, NK-cells, granulocytes and some related cells [119]. Elevated plasma IL-10 levels have been documented in type 1 and 2 diabetes patients as well as in degenerated intervertebral discs [64, 105]. In addition, IL-10 levels were up-regulated in rheumatoid arthritis and osteoarthritis models. This cytokine exerts anti-inflammatory, anti-catabolic as well as anti-apoptotic effects in chondrocytes, and is a potential target for curing arthritis [120]. The common characteristic of IVDD and osteoarthritis involves degradation of the extracellular matrix by regulating MMPs and other degrading enzymes to accelerate cell apoptosis [53, 121, 122]. Clinically, IL-10 inhibits the catabolic effects of pro-inflammatory cytokines by down-regulating MMPs and pro-inflammatory COX-2 [123]. IL-10 antagonizes matrix degrading enzymes and affects cartilage matrix gene expressions triggered by pro-inflammatory cytokines, such as TNF-α [123-125]. Behrendt reported that IL-10 significantly reduced the expressions of ADAMTS-4, MMP-3, and MMP-13, which were closely associated with ECM degradation, suggesting that IL10 has protective effects on chondrocytes [126]. Apoptosis significantly contributes to osteoarthritis and IVDD pathogenesis [33, 127]. IL-10 inhibits cell apoptosis by suppressing activated caspase-3 levels and the ratio of bax/bcl-2 to ameliorate the process of osteoarthritis. Moreover, it inhibits TNF-α-induced mitochondrial dependent apoptosis by increasing bcl-2 and down-regulating cleaved- caspase3 levels [123, 126]. Therefore, IL-10 has a significant role in interrelations between diabetes mellitus and IVDD.

**Figure 1.**
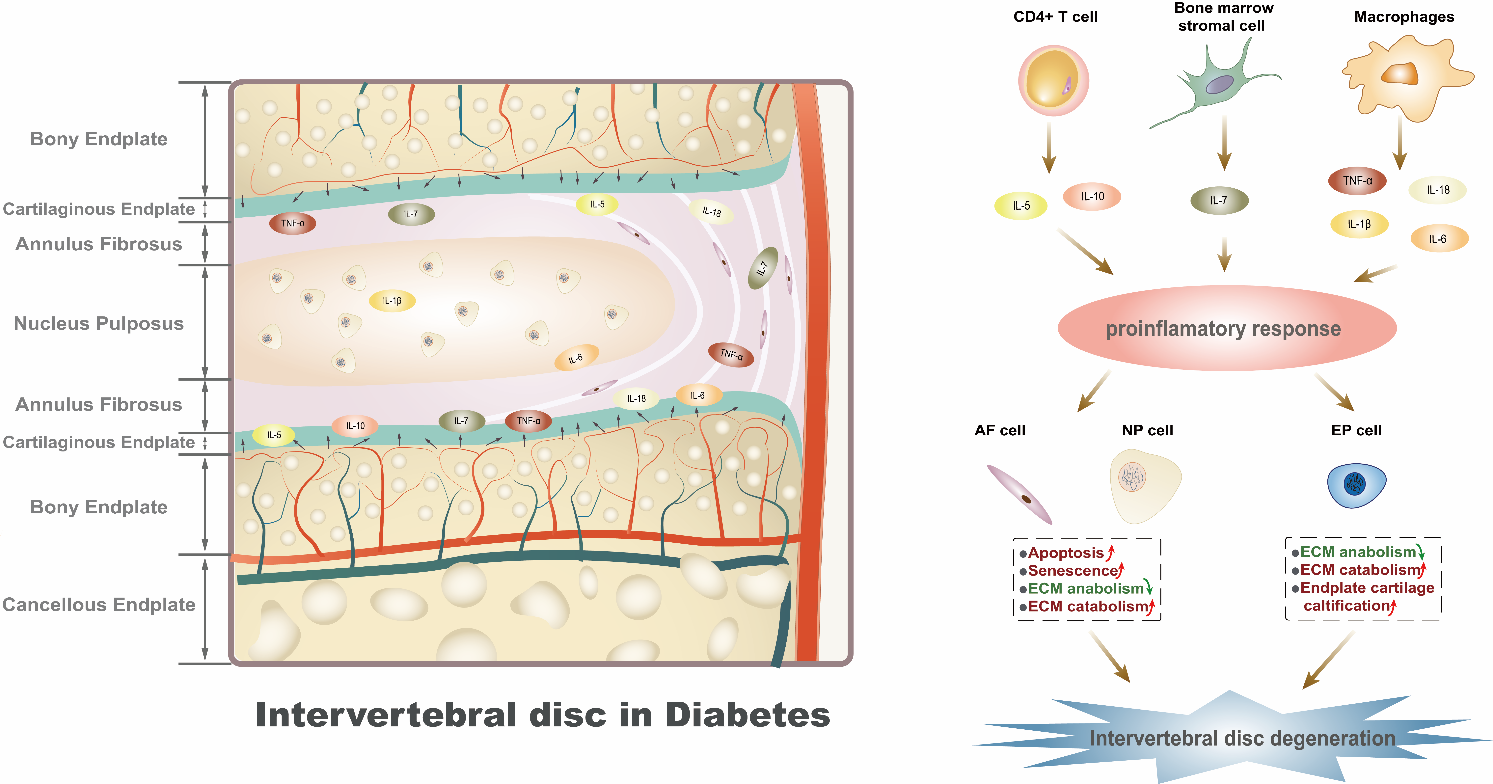
**The potential association between elevated cytokines and intervertebral disc degeneration in diabetic patients**. The plasma levels of IL-1β, IL-5, IL-6, IL-7, IL-10, IL-18, and TNF-α are elevated in diabetic patients, leading to intervertebral disc degeneration due to infiltration of the nucleus pulposus (NP) cells, annulus fibrosus (AF) cells, and endplate chondrocytes from endplate microvascular. Diabetes may alter the function of immune cells which release specific cytokines *in vivo* and augment inflammatory response. Such cytokines may contribute to IVDD by enhancing inflammation response, directly or indirectly regulate intervertebral disc via modulating the ECM anabolism and catabolism, apoptosis, and senescence.

## IL-18 in DB-IVDD

IL-18, a member of the IL-1 superfamily with a similar structure to IL-1β, is a highly regulated inflammatory cytokine that is cleaved by intracellular protease caspase-1 to generate a biologically active molecule. IL-18 has been reported to be elevated in inflammatory diseases and conditions such as, T2D, obesity, Alzheimer's disease, and ischemic heart disease [70, 128]. However, the function of elevated IL-18 in diabetes-induced IVDD remains unknown. T2D, obesity, and stress can promote the release of IL-18 from microglia. Moreover, IL-18 seems to increase ROS production in cells. The ROS in turn activates caspase-1 and inflammasome system leading to further production of IL-18 and neuronal apoptosis [129]. It has been shown that IL-18 can increase the protein level of anti-apoptotic BCL-2 and BCL-Xl, which are protective transmembrane proteins that inhibit the mitochondrial pathway of apoptosis in neurons [130]. A previous study revealed that IL-18 released from pyroptotic NPCs caused degeneration of the surrounding normal NPCs, thereby accelerating IVD degeneration [131]. IL-18 may also influence the endplate vascular endothelial cells, hence alter the environment around NP cells, AF cells, and endplate chondrocytes. The major pathological changes associated with IVDD include cartilage endplate degeneration and nucleus pulposus senescence or apoptosis. Calcification of the endplate cartilage is the major cause of endplate degeneration [20]. IL-18 can also induce inflammatory responses in synoviocytes and chondrocytes, and increase the expression of inflammatory factors, such as TNF-α, PGE_2_, and COX-2. In this way, it contributes to the cartilage degeneration and osteoarthritis [132]. Furthermore, studies have shown that IL-18 degrades the disc matrix and is elevated in serum of patients with IVD degeneration. Elsewhere, IL-18 up-regulated the expression of MMP13 and down-regulated the expression of anabolic factors such as Collagen II and SOX6 in human nucleus pulposus [133]. Therefore, IL-18 may play an important role in diabetes-induced IVDD, although the detailed mechanisms need to be further investigated.

It remains unclear how elevated cytokines in diabetes contribute to disc degeneration. Further research is advocated to reveal the mechanisms and develop novel treatments for disc degeneration targeting these cytokines.

## Conclusion

Diabetes has been reported to induce intervertebral disc degeneration. As the increasing prevalence of diabetes, diabetes induced IVDD is becoming a burning issue. However, the mechanisms involved in diabetes-induced IVDD have not been clearly illustrated. Inflammation, one of the main characteristics of diabetes, is the main pathogenic factor for various kinds of diseases, including IVDD. Herein, we summarized cytokines that are specifically elevated in diabetic condition, also we discussed the role of these cytokines in IVDD, including ECM metabolism, apoptosis, senescence as well as vascular ingrowth (Fig. 1). IL-1β and TNF-α have been reported to aggravate IVDD; therefore, inhibition of them is considered to be effective therapy for IVDD. However, other elevated cytokines such as IL-5, IL-6, IL-7, IL-10 and IL-18 may play different roles in IVDD, whose effects on IVDD are yet to be determined.
